# Predictors and (in-)stability of ICD-11 complex posttraumatic stress disorder in older adults: findings from a longitudinal study in Switzerland

**DOI:** 10.1080/20008066.2023.2299618

**Published:** 2024-01-23

**Authors:** Milan Rusmir, Shauna L. Rohner, Andreas Maercker, Aileen N. Salas Castillo, Myriam V. Thoma

**Affiliations:** a Psychopathology and Clinical Intervention, Institute of Psychology, University of Zurich, Zurich, Switzerland; b Competence Centre for Mental Health, Department of Health, OST – University of Applied Sciences of Eastern Switzerland, St. Gallen, Switzerland; c University Research Priority Program ‘Dynamics of Healthy Aging’, University of Zurich, Zurich, Switzerland

**Keywords:** Adverse childhood experiences, complex post-traumatic stress disorder, older adults, longitudinal study, emotion-related factors, Experiencias adversas en la infancia, trastorno de estrés postraumático complejo, adultos mayores, estudio longitudinal, factores relacionados con las emociones

## Abstract

**Objective:** There is a lack of research on complex post-traumatic stress disorder (CPTSD) in older individuals, with little known about the temporal course of CPTSD. Therefore, this study assessed and compared the demographic characteristics, adverse childhood experiences (ACE), and well-being of Swiss older adults with and without probable CPTSD. The (in-)stability of probable CPTSD was also examined in relation to the predictive value of various emotion-related factors.

**Methods:** A longitudinal study was conducted in Switzerland with *N* = 213 participants (*M*_age_ = 69.98 years, *SD* = 10.61; 45.5% female). Data was collected via face-to-face assessments at baseline and follow-up, 21 months apart. The German version of the *International Trauma Questionnaire* was used to screen for (C)PTSD. Standardized instruments were used to assess ACE as well as the predictors anger, embitterment, emotion regulation, and meaning in life.

**Results:** From the total sample, *n* = 16 participants (7.5%) were identified as having probable CPTSD, with only five of these (31.25%) having probable CPTSD at both baseline and follow-up. Individuals with and without probable CPTSD differed significantly regarding age and employment status. Significant predictors of probable CPTSD were anger (*β* = 0.16), embitterment (*β* = 0.06), cognitive reappraisal (*β* = −0.41), and the presence of meaning in life (*β* = −0.10).

**Conclusions:** Probable CPTSD appears to be relatively unstable over the course of a 21-month period in older individuals. The links between CPTSD and emotion-related predictors highlight potential targets for intervention.

## HIGHLIGHTS

- Key findings: Older adults with and without probable CPTSD differ in terms of age, employment status, adverse childhood experiences, psychiatric comorbidities, well-being, and life satisfaction.
- Temporal (in-)stability: In older adults, probable CPTSD appears to be unstable over a 21-month period with around one-third of the participants showing a stable course.
- Treatment targets: The identified significant predictors anger, embitterment, cognitive reappraisal, and the presence of meaning in life beliefs are potential treatment targets.

## Abstract (es)

**Objetivo:** Hay una falta de investigación sobre el trastorno de estrés postraumático complejo (TEPTC) en las personas mayores, y se sabe poco sobre el curso temporal del TEPTC. Por lo tanto, este estudio evaluó y comparó las características demográficas, las experiencias adversas de la infancia (ACE), y el bienestar de los adultos mayores suizos con y sin probable TEPTC. La (in)estabilidad del probable TEPTC también se examinó en relación con el valor predictivo de varios factores relacionados con las emociones.

**Método:** Se realizó un estudio longitudinal en Suiza con *N* = 213 participantes (Media de Edad = 69.98 años, DE = 10.61; 45.5% mujeres). Los datos se recopilaron mediante evaluaciones cara a cara al inicio y en el seguimiento, con 21 meses de diferencia. Se utilizó la versión alemana del Cuestionario Internacional de Trauma para detectar TEPT(C). Se utilizaron instrumentos estandarizados para evaluar las ACE, así como los predictores de ira, amargura, regulación de las emociones, y significado de la vida.

**Resultados:** De la muestra total, se identificó que *n* = 16 participantes (7.5%) tenían probable TEPTC, y solo cinco de ellos (31.25%) tenían probable TEPTC tanto al inicio como en el seguimiento. Los individuos con y sin TEPTC probable diferían significativamente en cuanto a edad y situación laboral. Los predictores significativos de probable TEPTC fueron la ira (*β* = 0.16), la amargura (*β* = 0.06), la reevaluación cognitiva (*β* = −0.41) y la presencia de significado en la vida (*β* = −0.10).

**Conclusiones:** El probable TEPTC parece ser relativamente inestable en el transcurso de un período de 21 meses en las personas mayores. Los vínculos entre el TEPTC y los predictores relacionados con las emociones resaltan objetivos potenciales para la intervención.

## 1. Introduction

Complex post-traumatic stress disorder (CPTSD) is a new diagnosis of ICD-11 (World Health Organization [WHO], 2019). It typically develops following exposure to persistent, repetitive, or multiple (usually interpersonal) traumas and/or victimization that has persisted for months or years, and from which escape is not possible or very difficult (Brewin, 2020; Maercker et al., 2022a). While a traumatic experience is a necessary causative element for CPTSD, it does not represent a sufficient criterion for its development (WHO, 2019). While most individuals experience some trauma across their life span, only a fraction of these will develop a specific, stress-related disorder in the aftermath of such experiences (e.g. de Vries & Olff, 2009). Current prevalence estimates for CPTSD range between 0.5% and 13% (Ben-Ezra et al., 2018; Cloitre et al., 2019; Hyland et al., 2020; 2021; Karatzias et al., 2019; Maercker et al., 2018). The discrepancy between the prevalence for traumatic experiences and stress-related disorders is reflective of the wide interindividual heterogeneity in post-traumatic stress responses and indicates that additional risk and protective factors must be considered.

Regarding potential socio-demographic risk factors, age and gender have previously been examined in relation to CPTSD. Regarding age, it is known that the emergence of CPTSD symptoms can happen at any age across the lifespan (WHO, 2019). While some studies reported younger age to be linked to CPTSD (Hyland et al., 2021; Karatzias et al., 2019), others did not (e.g. Maercker et al., 2018). Regarding gender, the existing data also appears to be inconclusive. While some research reported a higher risk for females (Cloitre et al., 2019; Kazlauskas et al., 2022), other studies found no meaningful link between gender and CPTSD (e.g. Hyland et al., 2021; Maercker et al., 2018). It may be that the relationships between age and gender with CPTSD are rather complex, as indications of a possible interaction between these two variables have also been previously reported (McGinty et al., 2021).

Additional demographic factors that have been linked to CPTSD are employment and relationship status. Studies have shown that individuals with (assumed) CPTSD (symptomatology) have a higher likelihood of being unemployed (Hyland et al., 2021), as well as being single, divorced, or widowed (Folke et al., 2019). However, Cloitre et al. (2019) found no meaningful links between CPTSD and employment or relationship status. With respect to socio-economic aspects, Kazlauskas et al. (2022) found that family financial difficulties (and loneliness) predicted (C)PTSD in adolescents. No differences regarding education have previously been reported (e.g. Cloitre et al., 2019). As such, while a number of studies have examined the link between various demographic characteristics and CPTSD, a clear picture has yet to emerge.

Another important risk factor to consider in CPTSD is the trauma profile, which has been studied in various large population samples. For instance, in a German nationwide sample, Maercker et al. (2018) found the highest rates of CPTSD to be linked to childhood sexual abuse or rape. Similarly, in a population-based study conducted in the United States (US), Cloitre et al. (2019) found cumulative childhood trauma and childhood (physical and sexual) abuse by caregivers to be linked to CPTSD. Furthermore, in a trauma-exposed, adult population sample in the United Kingdom (UK), Karatzias et al. (2019) found interpersonal trauma in childhood and adulthood to be linked to CPTSD. In Ireland, Hyland et al. (2021) reported that in addition to younger age and being unemployed; having been exposed to a higher number of traumatic experiences in childhood, adolescence, and adulthood; having an interpersonal trauma identified as their most distressing event (i.e. their index trauma); and fewer exposures to the index trauma were linked to CPTSD. As such, (a higher number of interpersonal) childhood trauma appears to be particularly relevant with respect to (the development of) CPTSD. It is important to note that childhood trauma and adverse childhood experiences (i.e. ACE; as assessed in the current study) are not fully interchangeable constructs. While both include childhood traumatic events (e.g. abuse and neglect), ACE also considers additional adversities and potentially traumatic factors, such as aspects related to household dysfunction and risk environments (see also Wingenfeld et al., 2011).

Studies on CPTSD that have been specifically conducted with older adults appear to be rare. One study by Krammer et al. (2016) was conducted with Swiss older survivors of child labour (mean age of 77 years). While childhood traumatic events were linked to some CPTSD symptoms, they were more strongly associated with PTSD symptomatology. However, in this study, CPTSD symptomatology was not assessed with the ICD-11 CPTSD criteria (Krammer et al., 2016). Another study with a clinical sample of Scottish older adults (mean age of 72 years) found that early maladaptive schemas mediated the relationship between traumatic childhood experiences and symptoms of CPTSD (Vasilopoulou et al., 2020). While there is a growing body of empirical evidence on the role of (cumulative, interpersonal) childhood trauma in the development of CPTSD, the link with CPTSD in older adults needs to be further explored. This is especially relevant as older adults are at risk of being undertreated. For instance, a study in the United Kingdom reported that older adults were significantly less likely to be referred for psychological treatment in comparison to younger adults (Pettit et al., 2017). Thus, although CPTSD can develop later in life, older people with CPTSD may experience chronic and potentially fluctuating symptoms that persist unrecognized and undertreated. It is therefore essential to examine if, similar to PTSD, there are age-specific differences in the symptoms, prevalence, or trajectory features of CPTSD (Pless Kaiser et al., 2019).

Regarding the severity of CPTSD, it is generally considered to be more severe, impairing, and enduring than PTSD (WHO, 2019). This is reflected by a heavier psychiatric burden (defined as symptoms of the major depressive and the generalized anxiety disorders) and a lower level of well-being (Cloitre et al., 2019). Regarding stability, a recent commentary on PTSD suggested that symptoms fluctuate more than accounted for by current diagnostic criteria (Fischer et al., 2023). However, apart from a few recent and valuable exceptions, only limited empirical knowledge exists regarding the temporal (in-)stability of CPTSD. One such study by Huang et al. (2023) assessed CPTSD three times over the course of six months in Chinese college students with ACE. The results identified three different, seemingly stable trajectories: A low-symptom group (41.8%), a moderate-symptom group (36.7%), and a high-risk group (21.4%) (Huang et al., 2023). Similarly, Dokkedahl et al. (2022) assessed (C)PTSD four times over the course of nine months in a Danish sample of females living in a women’s shelter. Results found a low- and high-symptom group, which both showed the biggest reductions in (C)PTSD symptoms within the first three months at the shelter and a stable trajectory thereafter. In a two-wave study with a general population sample in Israel, Hyland et al. (2020) found stable prevalence rates of CPTSD, PTSD, and Disturbances in Self-Organization (DSO) symptoms over the course of one year. While very limited empirical knowledge exists regarding the (in-)stability of CPTSD, the existing data points towards a decrease within the first months after the traumatic experience and a stable symptom course thereafter, as well as the potential existence of differential symptom groups. To the best of the authors’ knowledge, no study currently exists that observed CPTSD in older adults over a time period longer than one year.

In sum, with the relatively new developments in the ICD-11 CPTSD, and the small number of studies conducted with older individuals, little empirical knowledge exists with regard to CPTSD in older adulthood. However, knowledge on CPTSD in older individuals is of significant clinical relevance given the growing population of older adults; and that CPTSD is a common mental health disorder, which is linked to a higher psychiatric burden (i.e. more mental health symptoms) and lower well-being than PTSD (e.g. Cloitre et al., 2019; WHO, 2019). It was therefore the overarching goal of this study to examine CPTSD in a sample of Swiss older adults. In light of the relevance of childhood interpersonal trauma for the development of CPTSD, this study focused on the link between ACE and CPTSD in older individuals. Specifically, the first aim was to compare the demographic characteristics, ACE, and well-being of individuals with and without probable CPTSD. The second aim was to assess the temporal (in-)stability of probable CPTSD, by assessing CPTSD at two different time points, 21 months apart. To the best of the authors’ knowledge, this is the longest follow-up that has been examined thus far. Finally, given that difficulties in affect regulation is one of the most commonly reported reasons why individuals with a history of child maltreatment seek psychotherapeutic treatment (e.g. Schäfer et al., 2022); the third and explorative aim was to assess the link between various emotion-related predictors and probable CPTSD. The predictors anger, embitterment, emotion regulation, and meaning in life were chosen due to their previously established empirical link with PTSD, and/or ACE (e.g. Durham et al., 2022; Lee et al., 2017; Messman-Moore & Bhuptani, 2017; Spaaij et al., 2021).

## 2. Methods

Data for this longitudinal study was gathered within the Swiss project ‘Differential aging trajectories in high-risk individuals with past experiences of early adversity’, which was part of the National Research Program (NRP)76 ‘Welfare and Coercion – Past, Present, and Future’ (http://www.nrp76.ch/en) from the Swiss National Science Foundation (SNSF). The project consisted of a longitudinal (‘Main’) study and two mixed-methods sub-studies. Data used in this study was acquired in the ‘Main’ study, which was conducted from July to December 2019 (baseline assessment) and April to October 2021 (follow-up assessment).

The Ethics Committee of the Faculty of Arts and Social Sciences of the University of Zurich approved the original study protocol (ID: 19.4.3), as well as the amended protocol (ID: 20.12.24). The latter was necessary due to COVID-19 related adaptations to the study design (e.g. a three-month delayed start to the follow-up assessment, the implementation of protective measures). Written informed consent was provided by all participants and the study was conducted in accordance with the Declaration of Helsinki for research involving human participants. Baseline data from this project have previously been published (see Eising et al., 2021; Maercker et al., 2022b; Pfluger et al., 2021; Pfluger et al., 2022; Thoma, Bernays, Eising, Maercker, et al., 2021; Thoma, Bernays, Eising, Pfluger, et al., 2021; Thoma, Bernays, Pfluger, et al., 2021; Thoma et al., 2020).

### 2.1. Participants

The inclusion criteria required participants to be native Swiss-German speakers and have a minimum age of 50 years old. The sample consists of two groups: The ‘risk group’ (RG) consists of individuals who were affected by compulsory social measures and/or placements (CSMP) for a duration of at least one year during their childhood and/or adolescence. These participants had a high risk of being affected by childhood maltreatment and trauma (e.g. Krammer et al., 2016). The ‘control group’ (CG) is composed of age- and gender-matched individuals who were not affected by CSMP.

Participants in the RG were predominantly recruited using a list provided by the Swiss Federal Office of Justice, which was compiled when individuals affected by CSMP applied for a solidarity contribution. During this process, individuals could express their interest in participating in CSMP-related research studies. The contact information of the interested individuals was added to a list, which was provided to researchers conducting research within the NRP76 (Federal Office of Justice, 2023). Additional recruitment methods included contacting public figures who had experienced CSMP and through word-of-mouth recommendations. Individuals in the CG were recruited using flyers and advertisements distributed via websites and public spaces frequently visited by older adults (e.g. pharmacies, care homes). In addition, participants were recruited from a sample pool of the affiliated University Research Priority Program ‘Dynamics of Healthy Aging’ of the University of Zurich, Switzerland.

### 2.2. Study design

Those interested in participating in the study could contact the screening team who provided them with more details regarding the study. If the individuals showed continued interest, they were screened for the inclusion criteria. In the case that they fulfilled those criteria, two face-to-face baseline assessments (i.e. A1 and A2) were scheduled one week apart. Each appointment took a maximum of two hours and was conducted by trained interviewers. Prior to A1, participants were sent an information package containing the informed consent form, a detailed study description, and basic questionnaires concerning demographics and health. At A1, the informed consent was signed. For individuals in the RG, A1 started with an assessment of their CSMP experiences (e.g. age of first placement). A structured clinical interview assessing current and lifetime mental health disorders was then conducted with all participants. At the end of A1, participants received a package of questionnaires to be completed and returned at A2. The second assessment collected data on health, coping strategies, stress experiences, resilience resources, as well as a functional and cognitive tasks. After completing the baseline assessment, participants were reimbursed with 240 Swiss Francs and given a list of emergency and psychological counselling contacts.

The follow-up assessments (i.e. A3 and A4) were conducted 21 months later. At A3, (changes regarding) socio-demographic data were assessed. This was followed by a structured clinical interview to assess current and past year mental health disorders. In addition, stressful life events that participants may have experienced since the baseline assessment were assessed. Similar to the baseline assessment, participants were given a set of questionnaires at the end of A3 to be completed at home and returned at A4. The last assessment focused on cognitive and functional abilities, stress experiences, and psychological traits and states. Following the completion of A4, participants received 240 Swiss Francs and a list of emergency and psychological counselling contacts.

### 2.3. Measures

Standardized self-report questionnaires were used in German.

#### 2.3.1. Socio-demographic information

Socio-demographic information was gathered on age, gender, living situation, relationship status, level of education, employment category and status, and subjective satisfaction with current financial situation. This data was assessed at baseline and follow-up with the use of a self-report questionnaire.

#### 2.3.2. Adverse childhood experiences

To gather information regarding the number of different adverse childhood experiences, the *Adverse Childhood Experiences Questionnaire* (ACE; Wingenfeld et al., 2011) was used. The ACE contains ten dichotomous (yes/no) items, which assess potentially adverse or traumatizing events (e.g. physical or sexual abuse, separation from a parent) (total score range: 0–10). Higher scores reflect a higher number of different ACE. The CG completed the questionnaire once, whereas the RG completed the questionnaire twice: Once (if applicable) for the time they had lived with their family of origin (i.e. ‘ACE-family’), and once for the time they had experienced CSMP (i.e. ‘ACE-CSMP’).

#### 2.3.3. Psychiatric comorbidities

Psychiatric comorbidities were assessed according to DSM-5 with a shortened version of the *Diagnostic Interview for Mental Disorders* (DIPS; Margraf et al., 2017; Margraf et al., 2017). The following disorders were assessed: bipolar disorder, dysthymia, major depression, separation anxiety, panic disorder, generalized anxiety disorder, agoraphobia, social phobia, specific phobia, obsessive-compulsive disorder, acute stress disorder, somatic disorder, hypochondria, and psychotic symptoms. The score ranges from 0 to 19, with higher scores indicating more psychiatric comorbidities. Additionally, it was assessed whether the participants ever sought treatment for their potentially existing mental health problems (non-specific to any disorder).

#### 2.3.4. Well-being and satisfaction with life

Well-being of the past two weeks was assessed with the *World Health Organization Well-Being Index* (WHO-5; Brähler et al., 2007). It is composed of five items utilizing a Likert scale from 0–5 (total score range: 0-25). Higher scores indicate higher well-being.

Well-being relative to satisfaction with and quality of life was assessed with the *Satisfaction With Life Scale* (SWLS; Glaesmer et al., 2011). It is composed of five items utilizing a Likert scale from 1–7 (total score range: 5-35). Higher scores indicate higher satisfaction with life.

#### 2.3.5. (Complex) post-traumatic stress disorder

To assess whether participants fulfilled the ICD-11 criteria for probable (C)PTSD, the *International Trauma Questionnaire* (ITQ; Cloitre et al., 2018; Lueger-Schuster et al., 2018) was applied. It assessed six items for the core PTSD symptom clusters, six items for the DSO clusters, and six items for functional impairment. PTSD was fulfilled if the criteria were met for the core symptom cluster and functional impairment was present. CPTSD was fulfilled if all criteria were met for the PTSD core symptoms, DSO clusters, and functional impairment. As the ITQ is a self-report screening instrument, it does not allow for the assignment of a definitive diagnosis. To highlight this in the current study, participants who fulfilled the criteria for CPTSD or PTSD were described as individuals with (or without) a probable CPTSD or PTSD diagnosis. Individuals only fulfilling the DSO symptom cluster were also included to represent a potential subsyndromal form of CPTSD.

#### 2.3.6. Anger

Trait anger was assessed with the trait subscale of the *State-Trait Anger Expression Inventory* (STAXI; Schwenkmezger et al., 1992). The subscale consists of ten items utilizing a Likert scale of 1–4 (total score range: 10–40). Higher scores indicate a higher probable disposition towards anger.

#### 2.3.7. Embitterment

Embitterment was assessed with the self-report questionnaire *Berner Verbitterungs-Inventar* (BVI; Znoj, 2008). The BVI encompasses 18 items that are categorized into four subscales: Emotional embitterment, performance-related embitterment, pessimism, and misanthropy. The BVI uses Likert scales from 0 to 4 (total score range: 0–72). Higher scores indicate a higher level of general embitterment.

#### 2.3.8. Emotion regulation

Emotion regulation was assessed with the *Emotion Regulation Questionnaire* (ERQ; Gross & John, 2003). The ERQ encompasses ten items, with four items assessing expressive suppression and six items assessing cognitive reappraisal. It utilizes a Likert scale from 1 to 7 (total score range ‘suppression’: 4–28; ‘reappraisal’: 6–42). Higher scores indicate a higher use of the respective emotion regulation strategy.

#### 2.3.9. Meaning in life

Participants’ beliefs about the meaning in life were assessed with the *Meaning in Life Questionnaire* (MLQ; Steger et al., 2006). The MLQ contains ten items, with five items assessing ‘presence of meaning in life’ and five items assessing ‘search for meaning in life’. It utilizes a Likert scale from 1 to 7 (total score range: 5–35). Higher scores in each dimension indicate a higher presence of or search for meaning in life.

### 2.4. Data analysis

All analysis were performed using RStudio version 4.2.2 (RStudio Team, 2023). To compare demographic characteristics, a Pearson’s Chi-squared test was used for gender, and a two-sided Welch’s t-test was used for age. For the other categorical demographic characteristics, Fisher’s exact test was used as some expected values were smaller than five, which is a prerequisite for the Pearson’s Chi-squared test. For all group comparisons (i.e. CPTSD vs. non-CPTSD), a two-sided Welch’s t-test was used. These group comparisons were conducted for the analysis of ACE, psychiatric comorbidities, well-being, and satisfaction with life. Paired t-tests were used to compare the dimensional ITQ values at baseline and follow-up within the various symptom groups (CPTSD, PTSD, DSO, and none).

Missing data for the predictor values (anger, embitterment, cognitive reappraisal, expressive suppression, presence of and search for meaning in life) were imputed using the package ‘missRanger’, which uses a chaining random forests algorithm to impute mixed-type data sets. To impute the missing data, 100 trees were calculated and predictive mean matching was applied. The proportion of missing data was 2.8% or less on an item level. Univariable binary logistic regression analyses were first used for each predictor variable before calculating a multivariable binary logistic regression analysis using all predictors. McFadden, Cox–Snell, and Nagelkerke were the calculated R^2^ values. The Box-Tidwell test was used to check the linearity between the logit and the predictors. A significant *p*-value would indicate non-linearity, but all predictors were non-significant. The multicollinearity analysis using the variance inflation factor showed no values greater than 1.55, indicating only slight correlation between the predictors.

## 3. Results

### 3.1. Sample characteristics

The final sample consisted of *N *= 213 participants (mean age: 69.98 years, *SD *= 10.61, age range: 49–95 years). At either baseline or follow-up, 16 participants (7.5%) were identified as having probable CPTSD, ten participants in the RG, and six participants in the CG. Those with and without CPTSD differed significantly with respect to age (age range of those with CPTSD: 50-79) and employment status, but were comparable with respect to gender and the other socio-demographic variables (see Table 1).

**Table 1.** Sample characteristics.

	Total sample (*N *= 213)	CPTSD (*n *= 16)	Group comparison
Age (*M*, *SD*)	69.98 (10.61)	60.69 (9.90)	*t* = 3.8903 *p *= .001
Gender (female; %)	45.5	68.75	χ^2^ = 2.8139, *p *= .093
Living situation (%) Alone Partner Relatives Others Retirement home Miscellaneous	37.1 49.3 6.1 2.3 1.9 3.3	31.3 50.0 18.8 0 0 0	*p *= .415
Relationship status (%) Single In a relationship Married Separated Divorced Widowed	11.3 13.1 44.1 1.9 17.4 12.2	18.8 31.3 37.5 12.5 0 0	*p *= .165
Highest level of education (%) No education Primary school High school Vocational job training Higher professional training University Miscellaneous	2.3 3.3 51.2 15.5 23.0 4.7	0 0 68.8 18.8 12.5 0	*p *= .787
Job category (%) Agriculture Production Technical/IT Construction Commerce/traffic Hospitality Management/banking/law Health/teaching/science Miscellaneous	1.9 5.6 10.8 7.5 5.6 11.3 16.9 24.9 1.7	0 7.7 0 7.7 7.7 23.1 15.4 30.8 7.7	*p *= .826
Employment status (%) Employed Unemployed Retired Voluntary work	25.9 3.0 59.2 11.9	41.7 16.7 33.3 8.3	*p *= .001
Satisfaction with financial situation (*M*, *SD*)	2.86 (0.92)	2.50 (1.21)	*t* = 1.2678, *p* = .222

Note: *M* = mean, *SD* = standard deviation, CPTSD = complex posttraumatic stress disorder, χ^2^ = Pearson's Chi-squared test, *p *= *p*-value, *t *= two-sided Welch’s t-test.

### 3.2. Adverse childhood experiences

In a next step, the number of ACE were compared between those with and without CPTSD. Significant differences were found for the number of ‘ACE-family’ (*t* = −2.7588, *p *= .015): While individuals with CPTSD reported a mean of 4.71 ACE-family (range = 1–10), the non-CPTSD group reported a mean of 2.76 (range = 0-9). In the RG, no significant group differences were found regarding the number of ‘ACE-CSMP’ (see Table 2).

**Table 2.** Number of different adverse childhood experiences.

ACE-family		
**Non-CPTSD** (*n *= 168) *M* (*SD*)	**CPTSD** (*n *= 14) *M* (*SD*)	**Group comparison**
2.76 (2.41)	4.71 (2.55)	*t* = −2.7588 *p *= .015
ACE-CSMP		
**Non-CPTSD** (*n *= 89) *M* (*SD*)	**CPTSD** (*n *= 10) *M* (*SD*)	**Group comparison**
4.28 (2.27)	4.60 (2.99)	*t* = −0.3272, *p *= .750

Note*:* ACE-family = adverse childhood experiences experienced within the family of origin, ACE-CSM*P* = adverse childhood experiences experienced during compulsory social measures and placements, CPTSD = complex posttraumatic stress disorder, *M* = mean, *SD* = standard deviation, *t *= two-sided Welch’s t-test, *p *= *p*-value.

### 3.3. Psychiatric comorbidities, well-being, and life satisfaction

The total number of assessed psychiatric comorbidities was compared between those with and without CPTSD. The mean number of lifetime comorbidities was significantly higher in the CPTSD group (*M* = 3.06, range = 0–6), compared to the non-CPTSD group (*M* = 1.28, range = 0–7; *t* = −3.3427, *p *= .004). Out of the total sample, 120 participants sought treatment for their mental health. Within the CPTSD group, 13 individuals received professional mental health treatment.

With regard to the WHO-5 well-being sum scores, a significantly lower score of 9.33 (range = 1–19) was found in the CPTSD group, compared to the 16.11 (range = 1–25) in the non-CPTSD group (*t* = 4.3042, *p *< .001). Regarding life satisfaction total scores, a significantly lower score of 16.40 (range = 6–31) was found in the CPTSD group, compared to the 23.95 (range = 5–35) in the non-CPTSD group (*t* = 3.82, *p *= .002).

### 3.4. Temporal (In-)stability of CPTSD

Next, the temporal (in-)stability of probable CPTSD was examined. The number of participants who completed the ITQ at baseline and follow-up differed (*n* = 178 at baseline, *n* = 197 at follow-up). The majority (*n* = 26, 89.7%) of those who did not complete the questionnaire at baseline did not meet the criteria for probable CPTSD, PTSD, or DSO at the follow-up assessment. Three exceptions (from those who completed the questionnaire at follow-up, but not at baseline) must be mentioned: One participant was identified as having probable PTSD, and two participants as having DSO at follow-up. In addition, one participant who fulfilled the DSO criteria at baseline did not complete the ITQ at follow-up.

From the 16 participants who were identified as having probable CPTSD, five (31.25%) had probable CPTSD at both baseline and follow-up and were thus considered to be stable. Three participants (18.75%) showed a deterioration, which was defined as not having probable CPTSD at baseline, but then having probable CPTSD at follow-up. Eight participants (50%) showed an improvement, which was defined as having probable CPTSD at baseline, but not having probable CPTSD at follow-up. Similar results were found for probable PTSD and the DSO construct (see Table 3). Two special cases must be highlighted: One participant was identified as having probable CPTSD at baseline, and then probable PTSD at follow-up. Another participant showed the opposite picture. These two cases were categorized as improving and deteriorating, respectively, as CPTSD can be understood as a more debilitating disorder than PTSD.

**Table 3.** Stability of probable (complex) posttraumatic stress disorder and disturbances of self-organization.

Disorder *n* (%)	Total Baseline (*n *= 178)	Total Follow-up (*n *= 197)	Stable	Deterioration	Improvement
CPTSD	13 (7.3)	8 (4.1)	5 (31.3)	3 (18.8)	8 (50.0)
PTSD	9 (5.1)	10 (5.1)	1 (5.9)	8 (47.1)	8 (47.1)
DSO	14 (7.9)	10 (5.1)	6 (40.0)	2 (13.3)	7 (46.7)
None	142 (79.8)	169 (85.8)			

CPTSD: complex posttraumatic stress disorder; PTSD: posttraumatic stress disorder; DSO: disturbances of self-organization.

The ITQ not only allows for the assessment of probable (C)PTSD and DSO, but also includes dimensional total scores for these respective clusters. Table 4 lists the four subgroups of participants who were identified as having either probable CPTSD, PTSD, DSO, or none of the aforementioned clusters, separately for baseline and follow-up. Furthermore, the mean scores of the PTSD and DSO symptom clusters were compiled for each subgroup for baseline and follow-up. Two observations were significant: In the DSO subgroup, the DSO symptom scores were significantly lower at follow-up than at baseline (*t* = 2.215, *p *= .044), indicating an improvement in DSO symptom severity. Similarly, in the group without any probable (C)PTSD or DSO at any time point, the follow-up DSO symptom scores were also significantly lower than at baseline (*t* = 2.025, *p *= .045), indicating an improvement in DSO symptom severity.

**Table 4.** Dimensional International Trauma Questionnaire scores of the different symptom groups.

Disorder and symptomology scale (*M*, *SD*)	Baseline *n* = 178	Follow-up *n* = 197	Repeated measurement comparison
**CPTSD** PTSD DSO	16.25 (4.51) 13.69 (5.06)	12.75 (7.09) 11.63 (6.00)	*t* = 1.710, *p *= .108 *t* = 1.258, *p *= .228
**PTSD** PTSD DSO	13.12 (4.57) 7.24 (3.65)	11.67 (5.05) 6.18 (3.71)	*t *= 0.867, *p *= .399 *t* = 1.150, *p *= .268
**DSO** PTSD DSO	7.75 (3.84) 14.56 (4.88)	7.59 (3.86) 10.88 (5.53)	*t* = 0.330, *p *= .746 *t* = 2.215, *p *= .044
**None** PTSD DSO	3.88 (4.13) 3.37 (3.14)	3.27 (3.48) 2.84 (3.19)	*t* = 1.061, *p *= .291 *t* = 2.025, *p *= .045

Note: *M* = mean, *SD* = standard deviation*,* CPTSD = complex posttraumatic stress disorder, PTSD = posttraumatic stress disorder, DSO = disturbances of self-organization, *t *= paired *t*-test, *p *= *p*-value.

### 3.5. Predictors

Univariable binary logistic regression analyses assessed the effects of anger, embitterment, emotion regulation strategies, and meaning in life beliefs on the likelihood of having probable CPTSD at either baseline or follow-up (see Table 5). The univariable analyses revealed that anger (*β* = 0.16, *p* < .001), embitterment (*β* = 0.06, *p* < .001), cognitive reappraisal (*β* = −0.41, *p* = .043), and presence of meaning in life (*β* = −0.10, *p* = .004) were significant predictors for having probable CPTSD. An increase of one unit in the respective questionnaire scores was associated with an 18% increase for anger, a 6% increase for embitterment, a 34% decrease for cognitive reappraisal, and a 10% decrease for presence of meaning in life in the odds of having probable CPTSD. Expressive suppression (*β* = −0.04, *p* = .823), and search for meaning in life (*β* = 0.05, *p* = .095) were not significant predictors.

**Table 5.** Effects of various predictors on probable complex posttraumatic stress disorder.

Predictor	*β*0 (SE)	*β* (SE)	*p*	OR AOR	OR [95% CI] AOR [95% CI]	McFadden R^2^ Cox-Snell R^2^ Nagelkerke R^2^
Anger	−5.41 (0.94)	0.16 (0.05)	**	1.176 1.092	[1.076, 1.293] [0.982, 1.217]	0.110, 0.057, 0.138
Embitterment	−4.09 (0.59)	0.06 (0.02)	**	1.060 1.034	[1.029, 1.095] [0.993, 1.076]	0.128, 0.066, 0.160
Cognitive reappraisal	−0.71 (0.88)	−0.41 (0.20)	*	0.664 0.799	[0.446, 0.955] [0.510, 1.253]	0.035, 0.018, 0.044
Expressive suppression	−2.35 (0.76)	−0.04 (0.18)	.823	0.960 0.881	[0.668, 1.377] [0.582, 1.320]	<0.001, <0.001, <0.001
Presence of meaning in life	0.06 (0.87)	−0.10 (0.04)	*	0.900 0.961	[0.836, 0.967] [0.879, 1.050]	0.072, 0.037, 0.091
Search for meaning in life	−3.66 (0.79)	0.05 (0.03)	.095	1.052 1.016	[0.994, 1.122] [0.945, 1.098]	0.027, 0.014, 0.035

Note: *β*0 = intercept, *SE* = standard error, *β* = unstandardized beta, OR = odds ratio, AOR = adjusted odds ratio, CI = confidence interval, * = *p* < .05, ** = *p* < .001.

In an additional step to analyze the association of all predictors together, a multivariable binary logistic regression analysis was calculated. The analyses indicated that while testing one predictor and holding the others at a fixed value, none of the variables were significant predictors for having probable CPTSD. This would indicate multicollinearity which was checked with the Variance Influence Factor (VIF). Surprisingly, the VIF for any predictor did not exceed the critical subthreshold. The Hosmer-Lemeshow test was used to assess goodness-of-fit, which suggested a good model fit (χ^2^(8) = 11.57, *p *> .05) for the multivariable model. The model explained 22.4% (Nagelkerke R^2^) of the variance in probable CPTSD and correctly predicted 92.5% of cases.

## 4. Discussion

This study aimed to examine various characteristics related to ICD-11 probable CPTSD (PTSD, and DSO), as well as its temporal (in-)stability across almost two years, in Swiss older adults. Results found that older individuals with and without CPTSD differed with regard to age and employment status. Furthermore, individuals with CPTSD reported a higher number and higher frequency of different types of ACEs, more psychiatric comorbidities, a lower level of well-being, and lower satisfaction with life. In addition, results revealed that probable CPTSD appeared to be rather unstable over the 21 months. Finally, it was found that anger, embitterment, cognitive reappraisal, and the presence of meaning in life acted as meaningful predictors of CPTSD.

Individuals with probable CPTSD were significantly younger and had a higher probability to be unemployed, with less being retired. As employment status is dependent on age (i.e. retirement age in Switzerland is 65 years), it could be that these group differences are linked to the age differences. Nevertheless, being unemployed was also linked to CPTSD in previous studies (e.g. Hyland et al., 2021). The finding regarding individuals with probable CPTSD being younger also corroborates existing findings (Hyland et al., 2021; Karatzias et al., 2019). It also parallels PTSD findings, which suggest that higher age is linked to lower prevalence rates for PTSD. As such, younger and older age may be considered risk and protective factors, respectively. Individuals with and without probable CPTSD did not show gender differences, which is in line with existing research (e.g. Ben-Ezra et al., 2018; Maercker et al., 2018). It may be that CPTSD may not show the typical gender differences (i.e. higher prevalence for women), as commonly reported for PTSD, in a sample also consisting of individuals of higher age. However, given the small CPTSD sample in the current study, future research is needed to shed more light on potential gender differences in (older) individuals with CPTSD.

Regarding the experienced adversities, results showed that participants with probable CPTSD reported a higher number and higher frequency of different types of ACEs (i.e. emotional/physical abuse and emotional/physical neglect) than participants without CPTSD. The link between ACE and the later development of CPTSD may be explained with the recently developed cascade model of CPTSD by Maercker et al. (2022a), which proposes a sequence of connections between different factors (e.g. attachment, socio-interpersonal factors) in the relationship between ACE and the development of CPTSD. In a next step, future studies with older adults could examine these attachment and socio-interpersonal factors in the relationship between ACE and CPTSD.

Individuals with probable CPTSD had a higher number of psychiatric comorbidities across their life span, as well as a lower level of well-being and satisfaction with life. These findings are in line with previous reports from an US adult population study, which reported a heavier psychiatric burden and a lower level of well-being in individuals with CPTSD (Cloitre et al., 2019). As such, this study corroborates existing findings from younger samples by replicating them in a cohort of older adults.

The results of this study indicate that probable CPTSD, as assessed with the screening instrument ITQ, appears to be rather unstable over the course of 21 months. Only around one-third (31.25%) of the participants showed a stable course. Substantially more participants showed instability, either in the form of a deterioration or an improvement in symptoms. This is in contrast with previous reports of (apparent) stability in different symptom severity groups (e.g. Huang et al., 2023; Hyland et al., 2020); including one study that showed stability three months after the traumatic experience, following an initial decrease in (C)PTSD symptomatology (Dokkedahl et al., 2022). As such, this study extends the very limited literature on the temporal course of CPTSD by demonstrating that the majority of older individuals with CPTSD showed instability across two assessment points almost two years apart.

Regarding the predictors of CPTSD, higher trait anger and embitterment were linked to higher odds of having probable CPTSD. With respect to anger, this is consistent with previous findings by Murphy et al. (2021), which showed higher levels of anger in individuals with CPTSD in comparison to those with PTSD or none of these diagnoses. The embitterment results extend previous findings on the links between embitterment and (multiple) negative life events (e.g. You & Ju, 2020), or PTSD symptoms (Lee et al., 2021). This study further identified a significant negative link between emotion regulation (i.e. cognitive reappraisal) and probable CPTSD. These findings parallel results reported by Ehring and Ehlers (2014), which found meaningful links between emotion regulation and PTSD. Lastly, having a lower presence of meaning in life was linked to higher odds of having probable CPTSD. This builds on previous findings linking stronger meaning of life beliefs and fewer PTSD symptoms (Lee et al., 2021). However, in contrast to the study by Lee et al. (2021), which used a total score (comprised of the subscales), this study separately investigated the presence of and search for meaning in life subscales, which yielded a significant association only for the ‘presence’ subscale. Further research is needed to better understand these relationships and inform CPTSD treatment.

Several limitations of this study must be highlighted: First and foremost, this was a comparatively small sample and the number of individuals with probable CPTSD was particularly small, though the rate of individuals with probable CPTSD (7.5%) lies within the range of previously reported prevalence rates (Ben-Ezra et al., 2018; Cloitre et al., 2019; Hyland et al., 2020; 2021; Karatzias et al., 2019; Maercker et al., 2018). The small sample size may have led to a loss of power, particularly regarding the analysis of potential predictors. Therefore, these results should be viewed as preliminary. Furthermore, as the sample was self-selected, with half consisting of a very unique population of individuals, the generalization of these findings to other samples is restricted. The retrospective assessment of ACEs is an additional limitation, as it may result in recall biases. However, for older adults affected by the historical adversity of CSMP it was impossible to circumvent this bias. In addition, there was no information on (C)PTSD outside the study measurement points (e.g. when the CPTSD symptoms started) or whether participants received professional mental health treatment regarding their (C)PTSD symptoms. As such, it may be that some individuals, who improved from baseline to follow-up, may have been receiving therapeutical treatment for their symptoms. It must also be noted that between the two assessment points, the COVID-19 pandemic occurred, which may also have had an effect on the results. Additionally, probable CPTSD was assessed with a self-report measure (i.e. the ITQ), which may lead to an overestimation of CPTSD. Finally, several participants did not complete the ITQ at baseline, which may have led to a loss of information regarding potential (C)PTSD or DSO trajectories.

A major strength of this study is its longitudinal design spanning almost two years. This helped to significantly expand existing knowledge on the temporal (in-)stability of CPTSD. Another strength is the focus on older individuals, which is a population that is still much neglected in mental health research, particularly with respect to trauma-related disorders. Furthermore, the investigation of characteristics associated with CPTSD in older adults (e.g. emotion-related predictors) was a strength of this study as it can help to elucidate potential intervention targets in the treatment of CPTSD in older adults. In addition, the study provides insight into the (in-)stability of PTSD and the DSO symptom cluster.

Future research on associated characteristics and the temporal course of CPTSD (in older age) should apply larger sample sizes and longitudinal study designs. Concerning the emotion-related factors, there is also a need for appropriately powered, in-depth research on the respective predictors. The (potential) particular features of CPTSD in older age should also be empirically examined: According to ICD-11, the characteristics of CPTSD in older adults include physiological signs of anxiety, an anxious avoidance of triggers, and trauma survivors may feel great sorrow about how it has affected their lives (WHO, 2019). Additionally, subclinical levels or age-specific representations of CPTSD need continued investigation as these individuals could be in danger of being undertreated. Although the ITQ has previously been used to assess CPTSD in various nationwide studies that included older adults (e.g. Hyland et al., 2021; Maercker et al., 2018; McGinty et al., 2021), it has not yet been specifically validated for use with individuals of higher age. As such, future studies should aim to validate the ITQ for use in older adult samples. Furthermore, research is needed to examine mediators associated with a higher/lower vulnerability for the development of CPTSD. Studies should also examine the effectiveness of existing PTSD interventions in treating CPTSD, as well as the effectiveness of newly developed CPTSD interventions across various age groups.

### 4.1. Conclusion

With this study, it was possible to add novel empirical knowledge to the previously neglected research field of CPTSD in older individuals. It also added to the understanding of the temporal (in-)stability of CPTSD by assessing CPTSD at two-time points across a 21-month period. In light of the higher number of psychiatric comorbidities, as well as the lower levels of well-being and satisfaction with life, CPTSD appears to be a mental health disorder associated with a heavy burden for the affected individual. The identified predictors of CPTSD (i.e. trait anger, embitterment, cognitive reappraisal, and the presence of meaning in life beliefs) may provide relevant targets for therapeutic interventions. Given the small sample size of the CPTSD group, the novel findings of this study have to be interpreted cautiously and may be regarded as preliminary in nature. Nonetheless, these findings may provide a useful starting point in the quickly developing field of CPTSD (in older adults) to stimulate further research with older individuals.
